# The Growing One Century Club: Evolution of the Centenarian Population in Europe 2011–2021

**DOI:** 10.1093/geroni/igaf122.664

**Published:** 2025-12-31

**Authors:** Laetitia Teixeira, Lia Araujo, Oscar Ribeiro

**Affiliations:** ICBAS-UP, Porto, Porto, Portugal; RISE-Health, Porto, Porto, Portugal; University of Aveiro, Aveiro, Aveiro, Portugal

## Abstract

Longevity has been increasing in Europe, leading to changes in the distribution of centenarians across countries. This study analyzes changes in the distribution of centenarians across Europe between 2011 and 2021 based on data obtained from the CensusHub platform. Given the availability of data, 26 countries were included: Belgium, Bulgaria, Czechia, Denmark, Estonia, Ireland, Greece, Spain, France, Croatia, Italy, Cyprus, Latvia, Lithuania, Luxembourg, Hungary, Malta, Netherlands, Austria, Portugal, Slovenia, Slovakia, Finland, Sweden, Iceland and Norway. The number of centenarians per 100,000 inhabitants and the variation between 2011 and 2021 (in%) were calculated for each country, for the total population and by sex. The number of centenarians increased between 2011 and 2021. In 2011, the total number of centenarians was 57,951 (18.9 centenarians per 100,000 inhabitants) and in 2021 the number increased to 78,563 (25.2 centenarians per 100,000 inhabitants). In 2011, France, Italy, and Greece had the highest number of centenarians per 100,000 inhabitants, while in 2021, France, Italy, and Portugal led the ranking. Considering the variation (in %) in the decade, some countries revealed a negative variation: Lithuania (-35.4%), Hungary (-31.7%) and Iceland (-6.5%). The countries with the higher variations were Malta (100%), Ireland (92.2%) and Portugal (83.6%), both for total and for females. For males, higher variations were observed in Ireland (171.9%), Slovakia (108.2%) and Norway (93.1%); negative variations were observed in Lithuania, Hungary, Malta, and Cyprus. Although the overall number of centenarians increased, country-specific variations highlight the need for further research to understand underlying demographic and socio-economic factors.

